# Evaluating Electroencephalogram-Based Predictive Model for Drowsiness Measurement to Reduce Accident Risk in Active Individuals: Protocol for a Preliminary Monocentric Study

**DOI:** 10.2196/83969

**Published:** 2026-02-17

**Authors:** Chloé Boitard, Zoé Mazurie, Khadijeh Sadatnejad, Julien Coelho, Patricia Sagaspe, Julie Lenoir, Julien Mattei, Pierre Berthomier, Marie Brandewinder, Pierre Philip, Jean-Arthur Micoulaud Franchi, Christian Berthomier, Jacques Taillard

**Affiliations:** 1 Université de Bordeaux, SANPSY, UMR 6033, Groupe Hospitalier Pellegrin 13ème Etage – Aile 3 Place Amélie Raba Léon, Bordeaux, 33076, France, 33 05 57 82 01 72; 2 Centre Hospitalier Universitaire de Bordeaux, Service Universitaire de Médecine du Sommeil, Bordeaux, France; 3 Physip, Paris, France; 4 ENSAM, Paris, France

**Keywords:** cognitive performance, drowsiness, EEG, Objective Sleepiness Scale, prediction, sleep deprivation, electroencephalogram

## Abstract

**Background:**

Voluntary behaviors and socioeconomic factors, such as social jetlag and shift work, can lead to insufficient or disrupted sleep, resulting in drowsiness among active individuals. In occupational and driving contexts, drowsiness poses a serious safety risk by impairing alertness, slowing reaction times, and increasing the likelihood of accidents. Developing automatic and easy-to-implement tools for drowsiness detection or prediction is essential in the management of sleepy patients or in high-risk environments where sustained vigilance is critical.

**Objective:**

This study aims to validate continuous or predictive methods for assessing drowsiness using automated analysis of a limited number of electroencephalogram (EEG) channels.

**Methods:**

Designed as a single-center, nonrandomized, single-group study, this investigation will evaluate drowsiness and cognitive performance in 40 healthy volunteers exposed to 2 sleep deprivation conditions simulating real-world occupational scenarios. The primary outcome will be the Objective Sleepiness Scale (OSS) and its automated analysis, with a focus on its ability to measure objective wakefulness as assessed by the maintenance of wakefulness test (MWT). Secondary outcomes will include multimodal resting-state EEG markers, subjective and objective sleepiness measures, performance on a simulated driving task, attention, executive function, and vigilance assessments, as well as sleep quality, sleep quantity, and mind-wandering. The influence of sociodemographic and clinical variables on the measurement and prediction of drowsiness will also be systematically examined.

**Results:**

This study received funding from Physip and ANR (Agence Nationale de la Recherche, National Research Agency) in 2019, with ethical committee (Comité de Protection des Personnes, Committee for the Protection of Persons) approval in May 2022. Recruitment began in March 2023 and was completed in May 2025, with a database lock in June 2025. Data analysis started in June 2025 and is still ongoing.

**Conclusions:**

By validating these novel EEG-based measures, this study aims to lay the groundwork for proactive strategies for drowsiness management in occupational, transportation, and clinical settings.

## Introduction

Sleepiness is a physiological and behavioral “need state” or “need for sleep.” It plays a key role in the regulation of the sleep/wake cycle, especially in triggering sleep onset at the usual bedtime or during sleep deprivation. Sleepiness facilitates the transition from wakefulness to sleep and hinders the transition from sleep to wakefulness. “Physiological sleepiness” [1], also known as sleep drive, results from an imbalance between processes involved in the regulation of sleep and wake states. It is orchestrated both by the sleep homeostatic process, relying on the prior duration of wakefulness and the prior amount of sleep, and by the circadian rhythm, inducing a forbidden sleep zone at the end of the evening and an imposed sleep zone at the end of the night. Physiological sleepiness is modulated by interindividual differences (chronotype, age, and sex).

Manifest sleepiness [1] is the transformational effect of underlying physiological sleepiness on behavior due to voluntary or involuntary sleep deprivation, circadian misalignment, or pathologies inducing excessive daytime sleepiness. Manifest sleepiness is also referred to as drowsiness, hypoarousal [2], or continuous nonimperative sleepiness [3] by other authors. We will here use the generic term “drowsiness” to qualify manifest sleepiness. Drowsiness is characterized by an inappropriate waking behavior and a lasting inadequate level of arousal, which results in an inability to stay awake, difficulties in maintaining sustained attention and vigilance (ie, brain fog), and affects judgment and decision-making abilities and encourages risk-taking [4]. Drowsiness can thus induce functional consequences in terms of both disability and accident risk [5-7], to the same extent as the effects of alcohol consumption [8]. Drowsiness also redirects attention to thoughts that are not related to the task at hand [9]. This modification in attentional orientation, known as mind wandering, also impacts cognitive performance [9,10]. Finally, introspective sleepiness [1] concerns the individual’s self-assessment of their internal state of sleepiness. Manifest and introspective measures are linked because they both reflect underlying sleep pressure and drowsiness and therefore may arise from the same underlying drive state [11]. However, drowsy people often fail to accurately assess their level of drowsiness and therefore overestimate their ability to make important decisions and perform complex tasks [12], despite their decreased cognitive abilities, which can lead to dangerous consequences. Identifying and measuring real-time drowsiness or predicting it in high-risk situations like working or driving is thus a significant public health challenge [13,14].

Drowsiness can result from voluntary behaviors or socioeconomic factors that lead to insufficient or disrupted sleep [15], such as social jetlag [16] and shift work [17]. Drowsiness assessment over time is mainly based on brain activity measurements (electroencephalogram [EEG]) [18]. EEG provides a direct and objective window into neural correlates of alertness, unlike subjective measures or sporadic performance-based assessments. EEG data can reveal subtle transitions between wakefulness and drowsiness that may otherwise go undetected. Thus, the primary index of drowsiness, that is, the ability to stay awake, is assessed during the maintenance of wakefulness test (MWT) [19], an iterative electrophysiological test in which EEG and electrooculogram (EOG) analysis allow the determination of daytime sleep latency, identifying “global” daytime drowsiness. Moreover, the behavioral consequences of drowsiness —that is, impaired cognitive performance and reduced vigilance, characterized by slowed reaction times and an increased rate of commission errors (incorrect responses made when no response is required)—are associated with changes in EEG recordings, including episodic microsleep intrusions, local sleep phenomena, and wake-state instability [4,18]. Thus, EEG analysis, considered the “gold standard” for direct and continuous monitoring of sleepiness [18], represents the most promising way to detect drowsiness in safety-sensitive contexts, such as transportation, health care, and industrial operations, where lapses in vigilance can have serious consequences.

Given the need to provide continuous measurement to combat drowsiness in the workplace, the earliest EEG-based methods developed to continuously assess spontaneous drowsiness in offline conditions include the Karolinska Drowsiness Score [20]. This approach is designed to evaluate sleepiness-related impairments in performance, particularly in driving, by quantifying theta and alpha EEG activity and/or identifying specific slow rolling eye movements via EOG, resulting in a continuous sleepiness score ranging from 0 (not sleepy) to 100 (very sleepy). Currently, numerous EEG-based systems and algorithms—both offline and online—have been proposed for the detection of drowsiness. These methods often rely on various frequency band ratios, including theta/beta, beta/(alpha+beta), theta/(alpha+beta), (theta+alpha)/beta, (theta+ alpha)/(alpha+beta), and (gamma+beta)/(sigma+alpha) [14,21-23]. To enhance classification accuracy, some systems integrate additional physiological signals, such as electrocardiogram (ECG), EOG, electromyogram (EMG), respiratory activity, or behavioral data [14,23,24]. However, the practical application of these systems remains limited. Most have been validated only under laboratory conditions, typically during early afternoon sessions, whether after sleep deprivation or not, and their deployment in real-world settings is constrained by the large number of sensors required. To ensure operational feasibility, future development of EEG-based drowsiness detection systems should prioritize the use of a minimal number of sensors, particularly EEG derivations.

The Objective Sleepiness Scale (OSS), developed by Muzet et al [25], was designed to continuously monitor drowsiness and classify sleepiness into 5 levels based on the duration of alpha and/or theta EEG activity and the presence of slow eye movements, assessed over 20-second epochs. Despite its methodological clarity, the OSS is not widely adopted in subsequent research. However, a recent study has reported significant associations between OSS scores and sleepiness-related variables, such as sustained attention and driving performance [26], confirming the potential of the OSS to provide a reliable assessment of sleepiness-related cognitive impairments. The original implementation of the OSS required 4 EEG and 4 EOG derivations, which limited its applicability in operational settings. To address this, the French company Physip, in collaboration with Muzet et al [25], developed MEEGAWAKE, an algorithm for detecting and assessing drowsiness. Based on the OSS, the algorithm is capable of classifying the 5 stages of sleepiness, relying exclusively on EEG signals recorded from only 2 derivations. The performance of this algorithm was validated on 15 participants in a driving simulator by comparison with visual analysis conducted by a drowsiness reference expert [27]. This streamlined approach facilitates easier integration into workplace environments.

Nevertheless, current real-time spontaneous drowsiness detection systems continue to face several challenges. First, they typically require continuous wear by the operator, which complicates their implementation in real operational settings. Second, the detection of drowsiness often occurs too late to prevent its functional consequences. Therefore, the development of EEG-based tools allowing the prediction of drowsiness and its behavioral consequences—rather than detecting its presence—would mark a significant advancement in ensuring operator safety. In experimental protocols, routine EEG bio-calibrations, performed with eyes open and eyes closed, are critical for assessing signal quality and characterizing an individual’s resting-state brain activity. Resting-state EEG recordings, under both eyes-opened and eyes-closed conditions, have been used to evaluate task performance and drowsiness levels [28-31]. An algorithm developed by the University of Leipzig (VIGALL, The Vigilance Algorithm Leipzig) can identify 7 states of somnolence during a 5-minute resting-state EEG but requires the recording of at least 19 EEG leads (25 recommended), making it unusable in real operating conditions [32]. However, these short measures offer a promising avenue for prediction. Indeed, a brief, iterative, and easy-to-administer resting-state assessment could enable early identification of drowsiness and its potential cognitive and behavioral impacts, thereby facilitating timely and effective preventive interventions.

The objective of this study is to validate—under sleep deprivation conditions reflecting real-life occupational scenarios (such as delayed, fragmented, or disturbed sleep, typically experienced during night work, shift work, or on-call duty)—the ability of the OSS and its automatic analysis by the MEEGAWAKE algorithm to accurately assess drowsiness and its associated cognitive and behavioral consequences. In addition, the study aims to evaluate whether automatic analysis of resting-state EEG can reliably predict drowsiness and its functional outcomes, independently of the duration of prior wakefulness. Such predictive capability could enhance risk assessment and contribute to the prevention of sleepiness-related accidents in operational settings.

We hypothesize that both the OSS and resting-state EEG, potentially adjusted for interindividual variability in sleepiness susceptibility, can effectively measure and/or predict drowsiness and its behavioral consequences, including the inability to maintain wakefulness and impairments in sustained attention and vigilance, regardless of the time of day. This approach may provide a robust framework for identifying and mitigating risks associated with drowsiness in safety-critical environments.

## Methods

### Objectives

This study has 2 main objectives. This study’s first aim is to determine whether the OSS criteria can accurately detect manifest sleepiness, compared to the MWT, and to evaluate its temporal accuracy in detecting drowsiness-induced momentary behavioral outcomes, including driving performance, vigilance, and sustained and selective attention.

Second, this study aims to validate robust models based on a multimodal EEG index, derived from resting-state activity during bio-calibration, for predicting functional impairments linked to drowsiness, particularly in driving performance, vigilance, sustained, and selective attention.

### Study Design

This research is designed as a preliminary investigation with a single-center, nonrandomized, single-group study, conducted at the Sleep Department of the SANPSY laboratory, Bordeaux, France.

### Participants

#### Sample Size Calculation

This study is exploratory, since, to our knowledge, no other study has used the OSS to predict manifest sleepiness. Thus, because no data would allow us to statistically determine the number of participants required, this number has been arbitrarily set to 40 healthy volunteers. We believe this number will facilitate preliminary exploration at both inter- and intraindividual levels with an appropriate protocol.

#### Recruitment

Candidates are 40 healthy volunteers recruited from Bordeaux University Hospital’s healthy volunteer database and through advertisement via information flyers and internet recruitment. Participants were screened with respect to health status by a physician and filled out specific questionnaires with professional clinicians. The candidates were informed that they would receive financial compensation of €900 (US $1062.02) for participating in the whole protocol, and the physician provided full information about the study (design, potential risks and constraints, answers to potential questions) before collecting the volunteers’ informed, written, dated, and signed consent.

The eligibility criteria include the following: individuals (1) aged between 20 and 60 years, with a BMI between 18 and 27, (2) with good French skills and the ability to understand the study, and (3) who are nonprofessional drivers with a valid driver’s license (obtained at least one year ago). Individuals (1) experiencing severe psychiatric, neurological, or medical pathology or under psychotropic or cardiotropic drug treatments, (2) afflicted by chronic insomnia disorder, severe diurnal somnolence, or sleeping pathologies that can induce excessive daytime somnolence, (3) declaring substance dependency, alcohol abuse (>2 glasses/day) and/or excessive consumption of coffee, tea, or caffeine-based drinks (such as Coke, >5 cups/day), and (4) who perform night or shift work or being on care or on-call duty during the last 72 hours before the experimental sessions were excluded from the study.

Once included in the study, participants completed the following self-administered questionnaires, addressing specific items that serve as exclusion criteria for this study, namely the presence of depression and/or anxiety disorders, daytime sleepiness, and sleep-related disorders, such as obstructive sleep apnea syndrome (OSAS) and restless legs syndrome (RLS).

PHQ-4 (Patient Health Questionnaire) is a validated and reliable tool designed to reveal propensity for anxiety and depression [33]. Participants are asked to score 4 items reflecting their tendency to feel anxious (2 items) or depressed (2 items) during the previous 2 weeks (on a Likert scale ranging from 0 (never) to 3 (every day). A score above 2 on any item or a total score above 4 on the anxiety or depression items will lead to the participant's exclusion.

ESS (Epworth Sleepiness Scale) is a common measure used to assess daytime sleepiness [34]. Candidates must score their ability to fall asleep in 8 different daily situations over the past months (on a Likert scale ranging from 0 (never) to 3 (very high probability). A score over 11, reflecting a severe diurnal somnolence, will lead to the participant’s exclusion.

STOP-BANG (snoring, tiredness, observed apnea, blood pressure, BMI, age, neck circumference, and gender) questionnaire evaluates the risk of OSAS through several items [35]. A score over 5, reflecting a high probability of being diagnosed with OSAS, will lead to the participant’s exclusion.

The RLS questionnaire assesses the risk of being diagnosed with this syndrome through 7 specific binary questions (yes or no answer) [36]. A positive answer to some items, reflecting a restless legs syndrome suspicion, will lead to the participant’s exclusion.

Finally, before the selected candidates entered experimental procedures, additional questionnaires were used to assess their chronotype and tendency to daydream (Table 1)(Table 1).

The chronotype was assessed using the 19-question Horne and Östberg Morningness/Eveningness questionnaire, which investigates life preferences (activity, wake/sleep cycle, and meal), somnolence, and tiredness at certain times of the day [37]. Scores vary from 16 to 86 and, with adaptation to the individual’s age [38], allow for the identification of evening persons (score ≤42 for those aged 20‐44 years and ≤53 for participants aged 44‐60 years), morning persons (score ≥58 and≥64, respectively) and extreme chronotypes (score ≤31 and ≤47 respectively, indicating highly evening persons, and score ≥69, indicating highly morning persons regardless of age). The French version [39] of the Munich Chronotype Questionnaire [40] was also used. It is composed of 7 questions investigating sleep habits on working days and 7 questions investigating sleep habits on free days. The sleep-corrected local time of midsleep on work-free days (MSFsc) and midsleep on working days (MSWsc) [40] will be identified, and the sleep-corrected social jetlag will be calculated (MSFsc – MSWsc) [41].

The candidates’ tendency to daydream in everyday situations was assessed using the Daydreaming Frequency Scale (DDFS) [42]. Participants are asked to rate their tendency to daydream during 12 common situations on a 5-point Likert scale (from 1 [very rare] to 5 [very frequent]).

After verification of inclusion and exclusion criteria, participants went through the experimental schedule, which was composed of 2 experimental sessions. Both sessions were designed to increase the sleep pressure through alteration in sleep quantity, while mimicking real-life work experiences. Each candidate’s participation lasted 14-35 days, according to the time lapse between the 2 experimental sessions (refer to Table 1). All premature withdrawals (eg, withdrawal of consent, inability to complete the protocol for medical or logistical reasons) will be documented, including date, reason, and any data collected up to the point of discontinuation. Unless a participant requests complete removal of their data, baseline characteristics and available measurements will be retained for analysis. The study aims to include 40 participants completing the full protocol. Replacement of withdrawals may be performed, if necessary, but will be limited to avoid overselection of highly motivated individuals (will not exceed 20% of the initial target sample size). Baseline characteristics of completers and noncompleters will be summarized and compared, along with a brief description of the reasons for withdrawal. Exploratory analyses may assess whether baseline factors predict early withdrawal. Primary analyses will be conducted on participants who complete the full protocol (per-protocol analysis). When feasible, data from partial completers will be included in exploratory models to maximize the use of available information. Mixed-effects models or other appropriate statistical approaches will handle repeated measures and missing data due to withdrawal. Participant flow (screened, enrolled, completed, and withdrawn) will be reported in a CONSORT (Consolidated Standards of Reporting Trials)-style diagram, and all analyses related to dropouts will be documented.

**Table 1.** Schedule of participants’ enrollment and assessments.

		Study period				
		Interview	Enrollment	Experimental schedule		Close-out
Timepoint		T−1 to −15 days	T0	Session 1: T+1 to T+14 days	Session 2: T+7 to T+30 days	T+7 to T+30 days
Recruitment						
Information about the study		✓				
Eligibility screen		✓	✓			
Informed consent			✓			
Clinical examination			✓			
Inclusion self-questionnaires (PHQ-4^a^, ESS^b^, STOP-BANG^c^, RLS)^d^			✓			
Individual characteristics (chronotype, daydreaming)			✓			
Assessments						
Actimetry				✓	✓	
Polysomnography				✓	✓	
Continuous wake EEG^e^ recordings (OSS^f^, resting state EEG)				✓	✓	
Karolinska Sleepiness Scale				✓	✓	
Driving simulation				✓	✓	
Maintenance of wakefulness test				✓	✓	
Cognitive tests				✓	✓	
Conscient Experience Characterization				✓	✓	

a PHQ-4: Patient Health Questionnaire-4.

b ESS: Epworth Sleepiness Scale.

c STOP-BANG: snoring, tiredness, observed apnea, blood pressure, BMI, age, neck circumference, and gender.

d RLS: Restless Legs Syndrome.

e EEG: electroencephalogram.

f OSS: Objective Sleepiness Scale.

### Experimental Timeline

**Figure 1.**
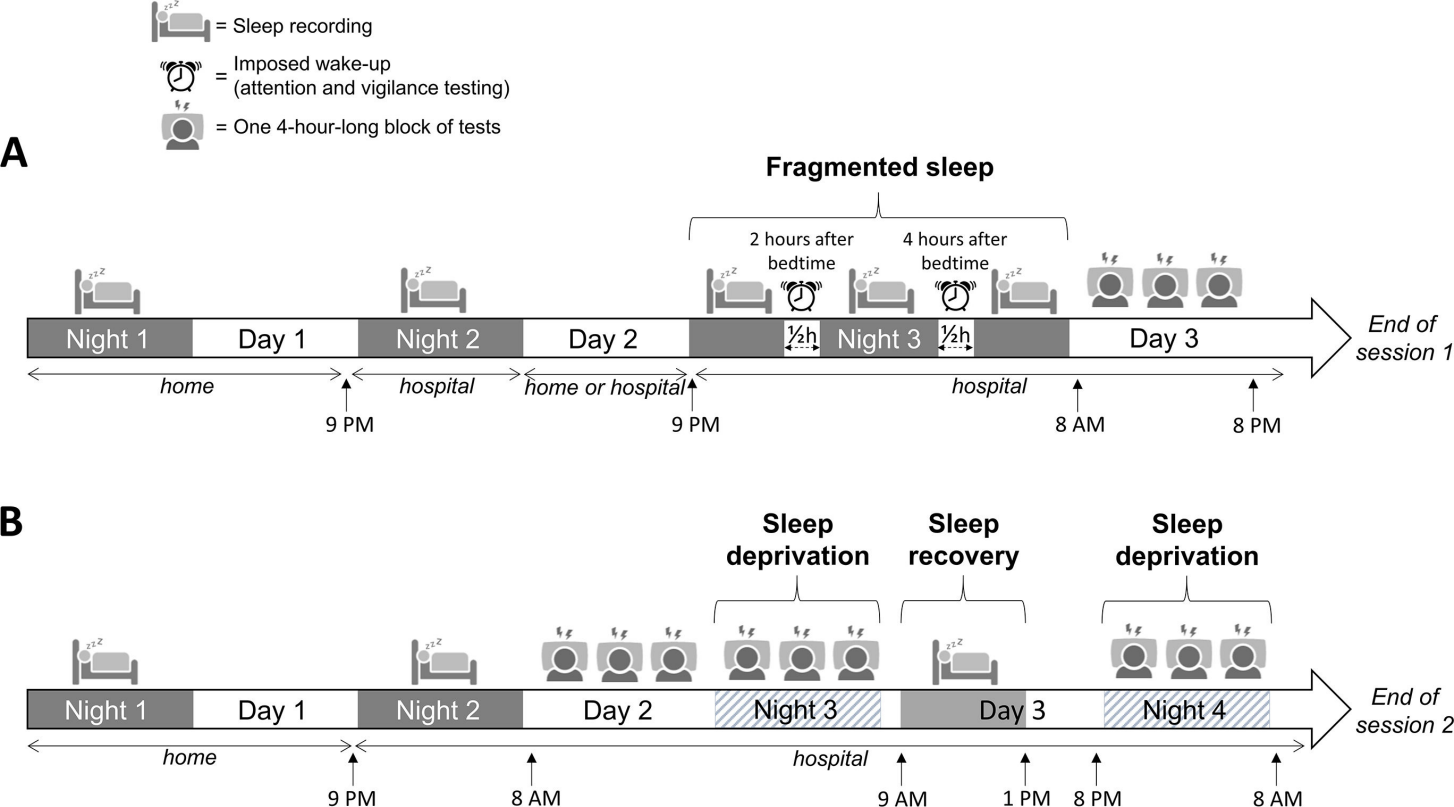
Study experimental workflow. (A) Experimental session 1 designed to create sleep deprivation conditions that simulate on-call duty. (B) Experimental session 2 designed to produce sleep deprivation conditions simulating 2 successive working nights.

The experimental schedule was composed of 2 experimental sessions, both designed to increase sleep pressure by altering sleep quantity prior to behavioral testing. Each candidate participated in both sessions in the same order. The first session lasted 3 days and was structured with a fragmented sleep schedule to simulate nocturnal on-call duty. The second session lasted 4 days with 2 full nights of sleep deprivation, separated by a 4-hour recovery period, to simulate a night shift (Figure 1). As shown in Figure 1, gray rectangles indicate periods of recorded sleep, white rectangles correspond to wake periods, hatched rectangles indicate night sleep deprivation.

**Figure 2.**
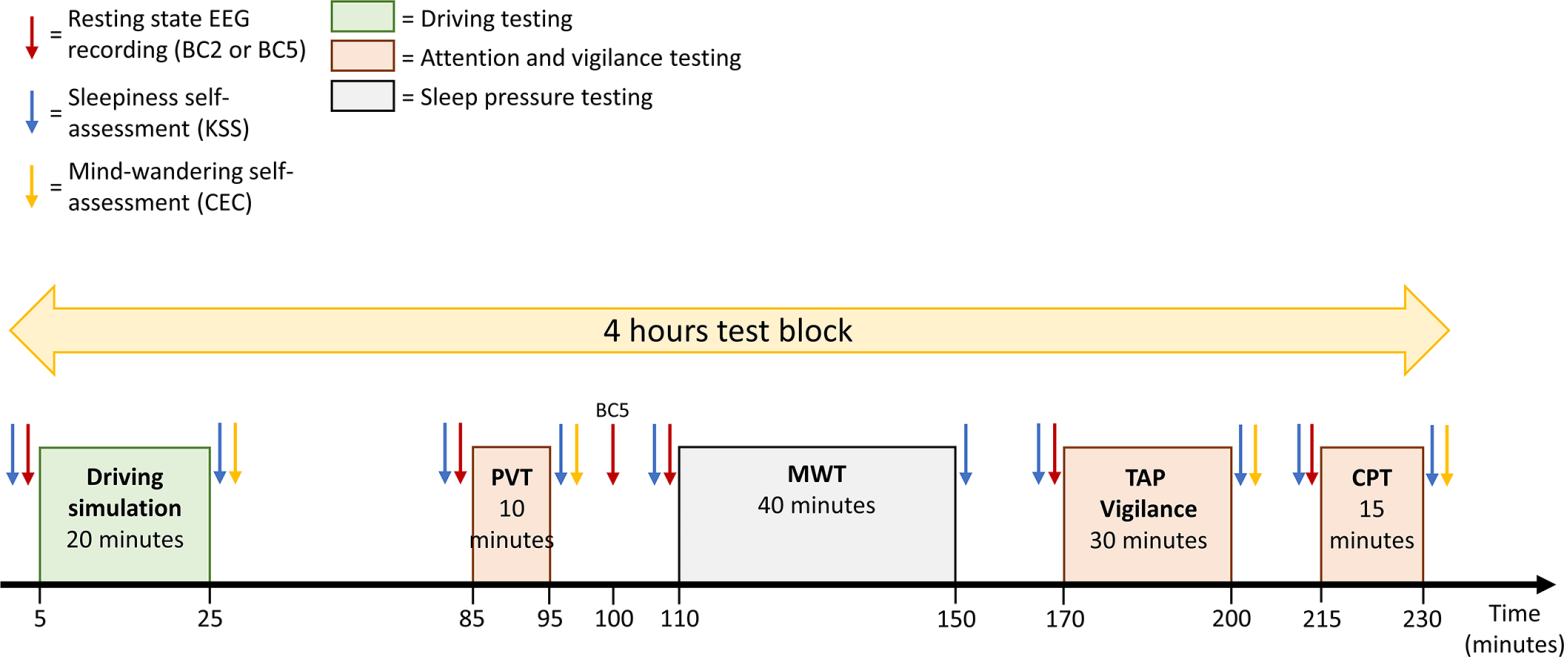
Timeline of one block of tests and questionnaires. CPT: continuous performance test; CEC: Conscient Experience Characterization; EEG: electroencephalogram; KSS: Karolinska Sleepiness Scale; MWT: maintenance of wakefulness test; PVT: psychomotor vigilance task; TAP: Test of Attentional Performance; BC5: 5-minute bio-calibration.

Several behavioral tests, with established validity for assessing sleepiness and its impact on cognitive performance (manifest sleepiness), described in the following section, were conducted during both sessions to assess the effects of sleep alteration on introspective sleepiness, on the capacity to remain awake (reflecting sleep pressure), on driving performance, on attention and vigilance, and on the mind-wandering experienced during the tests (Figure 2). This figure shows colored arrows, which indicate, before and/or after each test, the registration of the resting state EEG (bio-calibration lasting 2 minutes [BC2], red arrows) and the scoring by the participant of their introspective sleepiness level (KSS, blue arrows) and of their mind wandering during the test (CEC, yellow arrows). The pause from 25-85 minutes allows lunch or dinner to be served to the participants during the relevant blocks (starting at noon and 8 PM). Colored rectangles correspond to the different tests composing the 4-hour test block.

EEG was recorded throughout the entire experimental protocol, during the rest periods to assess sleep duration and quality in both ambulatory and laboratory conditions, and throughout the tests to allow for drowsiness assessment (through OSS visual and automatic scoring). Resting state EEG was recorded during regular bio-calibrations (BC2 or BC5 lasting 2 or 5 minutes, respectively).

### Sleep and Wake Monitoring Setup

Sleep parameters were assessed using ambulatory and laboratory polysomnography coupled with actigraphy. Activity was monitored using a wrist-worn accelerometer (GT9X Link, ActiGraph). Ambulatory polysomnography was performed using an EMBLA Titanium system (Natus; sampling frequency 256 Hz; 16-bit resolution). The montage included 2 EEG channels (Cz and Pz). Laboratory polysomnography was recorded with an Embla NDx amplifier and stored using Natus SleepWorks software (sampling frequency 256 Hz; 16-bit resolution). The montage included at least 2 EOG channels, 1 EMG channel, 6 EEG channels (F4, C4, O2, Cz, Pz, and A1), and 1 ECG channel.

While awake, participants were equipped with a gel-based active electrode system for EEG (Acticap Slim, Brain Products) connected to a compact wireless EEG amplifier LiveAmp (Brain Product: 24-bit resolution). The montage included 8 EEG derivations, namely Fz, Cz, Pz, C4, O2, C3, O1, and P3, and 4 vertical and horizontal EOG. All signals were acquired using a sampling frequency of 500 Hz.

### Description of the Tests

Introspective sleepiness was investigated through the Karolinska Sleepiness Scale (KSS), a subjective scale assessing the level of sleepiness during the past 10 minutes [43]. Participants are required to rate their ability to maintain wakefulness on a Likert scale (from 1 = “perfectly awake” to 9 = “severely somnolent, cannot stay awake”). This score is known to correlate well with EEG measurements and cognitive and behavioral performances linked to the candidate’s ability to maintain wakefulness [44].

The ability to stay awake under sleep-promoting conditions was monitored through the MWT, which is considered the gold standard in the measurement of manifest sleepiness [18,45]. The MWT is a 40-minute trial during which the participant is in a comfortable position in bed, in a quiet, dimly lit room. At the beginning of each trial, the candidate is instructed to stay awake and combat against sleep, to keep their eyes open, and to look straight ahead, without looking at the light. During the test, visual analysis of the polysomnographic recording allows for monitoring the participant’s state, and the test is stopped when unequivocal sleep is detected (defined by 3 consecutive epochs of stage N1 sleep or 1 epoch of any other sleep stage, according to the American Academy of Sleep Medicine (AASM) recommendations [46]).

Driving performance was assessed during a driving simulation procedure, as it provides an ecological approach that is commonly used to assess the effects of sleepiness on complex cognitive performance and safety, using the fixed-base INRETS-MSIS SIM2 (Institut National de Recherche sur les Transports et leur Sécurité — Modélisations, Simulations et Systèmes d’Information pour la Sécurité — Simulateur version 2; National Institute for Transport and Safety Research — Modeling, Simulation, and Information Systems for Safety — Simulator version 2) driving simulator [47]. In healthy individuals, this simulator has been shown to effectively assess nocturnal driving impairment in a dose-response design, considering extended wakefulness and driving duration, compared to real driving conditions [48]. The driving simulator is composed of a computer and a video game steering wheel with no force feedback applied. The participant’s head is positioned at 60 cm in front of the screen. The resolution of the visual scene was 1024×768 pixels, and the update rate was 60 Hz. The simulator generates a highway driving scenario on a 19-inch screen. The car’s speed was fixed by the experimenter at 130 km/h, and participants were instructed to drive in the center of the right lane. The test lasts for 20 minutes, during which the participant must maintain the car’s trajectory on a monotonous 2-lane highway (reconstruction of the French A62 highway, without any scenery or vehicles).

To evaluate the impact of sleepiness on cognitive performance of increasing complexity, we included 3 tasks ranging from basic attentional measures to more demanding cognitive functions. First, the participants completed the Psychomotor Vigilance Task (PVT), a widely used and validated tool for quantifying manifest sleepiness, particularly through its effects especially on sustained attention and somewhat less on vigilance [18]. The test consists of a one-choice serial reaction time task and was conducted using PC-PVT software (Biotechnology HPC Software Applications Institute), a freely available package for PVT testing, analyzing, and visualizing on a PC, whose performance is comparable to the PVT-192, the gold standard for measuring PVT [49]. The PC-PVT software [50] was installed on a desktop computer running under Windows 10 and equipped with a mouse. The test session lasts 10 minutes, during which a visual stimulus randomly appears on a screen, with interstimulus intervals varying from 2 to 10 seconds. The participant is asked to react as quickly as possible (by a mouse click) to make the stimulus disappear.

The Conners continuous performance test II (CPT-II) is a Go/No-Go task that evaluates the participant’s ability to maintain sustained attention and to discriminate relevant stimuli [51]. The test assesses inhibitory control, which is known to be highly sensitive to excessive daytime sleepiness [52-54]. The CPT (continuous performance test) paradigm is based on monitoring and responding to regular targets (here, all alphabet letters except the “X”), while withholding the response to a specific infrequent nontarget (letter “X”). CPT software (Multi-Health Systems Inc, version 5.2) was installed on a desktop computer running under Windows 10 and equipped with a keyboard. The test session lasts for 15 minutes, and the participant is asked to push the space bar when any letter is displayed on the screen, except for the letter “X” (target stimulus).

The Vigilance Test of Attentional Performance (TAP) is a rarely used animated line test, which measures the ability to focus and to maintain mental effort and vigilance on a monotonous task during a 30-minute period [55]. The test displays a horizontal line going up and down on a screen at an irregular speed, and from time to time, this line oscillates with a greater amplitude. TAP Vigilance (Psytest) software was installed on a desktop computer running under Windows 10 and equipped with a specific external response button. The participant is asked to push the response button when they notice a difference in the line oscillation amplitude (target).

Finally, mind-wandering during completion of the test was assessed through a subjective scale, the Conscient Experience Characterization (CEC) [56]. At the end of the cognitive tests, participants were asked to rate their focusing level during the previous task, using a scale of five options: (1) fully focused (attention and thoughts entirely dedicated to the task), (2) focus interference related to the task (attention and thoughts distracted by task characteristics or by their performance), (3) external distraction (attention and thoughts distracted by environmental stimuli with no relation to the task), (4) mind wandering (not focused, with thoughts disconnected from the task or the environment), and (5) empty mind (not focused, without any particular thoughts).

### Experimental Session 1: Fragmented Sleep

This session lasted for 3 consecutive nights and days, during which sleep was recorded. In order to induce sleep pressure on day 3, the third night was fragmented (with 2 imposed wake-ups, during which attentional and vigilance tests were carried out). Three blocks of behavioral tests were conducted during the third day (Figure 1A). One block of tests is 4 hours long and includes, in a fixed order, a driving simulation, cognitive tests, evaluation of sleep pressure, and self-assessments of drowsiness and mind-wandering, while continuous EEG is recorded (Figure 2).

The detailed schedule of days and nights is organized as follows:

1. Night 1: In the evening before the first night, the participants arrived at the laboratory to be equipped with Ag-AgCl electrodes (2 EEG derivations: Cz and Pz, connected to the EMBLA Titanium) to measure their sleep quantity and quality. They returned home for the first night, went to sleep, and woke up according to their usual schedule.
2. Day 1: After waking up and removing the sleep recorder, the participants carried out their usual activities throughout the day before returning to the laboratory in the evening, where training for the cognitive tests and driving simulation was provided.
3. Night 2: The participants were equipped with Ag-AgCl electrodes (6 EEG derivations: F4, C4, O2, Cz, Pz, A1, 1 EMG, 2 EOG, and 1 ECG) connected to the Embla NDx amplifier to measure their sleep quantity and quality. They spent the night in the laboratory, went to sleep, and woke up according to their usual schedule.
4. Day 2: After waking up, the participants either stayed in the laboratory or went to their regular activities before returning in the evening.
5. Night 3: The participants were re-equipped with polysomnographic sensors (as during night 2) and went to bed by midnight. They were awakened twice during the night (2 hours and 4 hours after bedtime) for 30 minutes, during which they underwent the PVT and CPT tests to measure their vigilance along with their ability to maintain sustained attention.
6. Day 3: After waking up at 7 AM, the equipment was removed, and the participants were equipped with a gel-based active electrode system for EEG. The participants underwent 3 test blocks. Block 1 occurred from 8 AM to noon, block 2 from noon to 4 PM, and block 3 from 4 PM to 8 PM After the last block, the EEG helmet was removed, and the participants either stayed in the laboratory for a recovery night or left the hospital, if accompanied, with a recommendation to go home for a recovery night.

### Experimental Session 2: Sleep Deprivation

This session lasted for 4 consecutive nights and 3 days, during which sleep was recorded. During the second day, the participants began a series of six 4-hour test blocks, lasting 24 hours and leading to a total sleep deprivation during the third night. On the third day, the participants had a morning recovery sleep period (4 hours) and began a new night of total sleep deprivation with a series of three 4-hour test blocks during the fourth night (Figure 1).

The detailed schedule of days and nights is organized as follows:

1. Night 1: In the evening before the first night, the participants entered the laboratory to be equipped with Ag-AgCl electrodes (2 EEG derivations: Cz and Pz) connected to the EMBLA Titanium to measure their sleep quantity and quality. They returned home for the first night, went to sleep, and woke up according to their usual schedule.
2. Day 1: After waking up and removing the sleep recorder, participants carried out their usual activities throughout the day before returning to the laboratory in the evening.
3. Night 2: The participants were equipped with Ag-AgCl electrodes (6 EEG derivations: F4, C4, O2, Cz, Pz, A1, 1 EMG, 2 EOG, and 1 ECG) connected to the Embla NDx amplifier to measure their sleep quantity and quality. They spent the night in the laboratory and went to bed by midnight.
4. Day 2 and Night 3: After waking up, the equipment was removed, and the participants had breakfast before being equipped with a gel-based active electrode system for EEG. Then, the participants completed six 4-hour test blocks. Block 1 occurred from 8 AM to noon, block 2 from noon to 4 PM, block 3 from 4 PM to 8 PM, block 4 from 8 PM to midnight, block 5 from midnight to 4 AM, and block 6 from 4 AM to 8 AM. After the last block, the EEG helmet was removed.
5. Day 3: The participants underwent a morning recovery sleep period (from 9 AM to 1 PM) during which they were equipped with the Ag-AgCl electrodes and sleep recorder, as described for night 2, to measure their sleep quantity and quality. After waking up, sensors were removed, and the participants remained in the laboratory without sleeping during the afternoon.
6. Night 4: The participants were re-equipped with the gel-based active electrode system for EEG and wireless EEG amplifier, as described for Day 2 and Night 3, and underwent a new series of three 4-hour test blocks. Block 1 occurred from 8 PM to midnight, block 2 from midnight to 4 AM, and block 3 from 4 AM to 8 AM After the last block, the EEG helmet was removed.
7. Day 4: The participant either stayed in the laboratory for a 5-hour recovery sleep or left the hospital, if accompanied, with a recommendation to plan a sleep recovery night at home.

### Protocol Assistant

Protocol Assistant is a software tool developed by Physip to manage the timeline of the various behavioral tests and questionnaires during the 4-hour block and to ensure synchronization with the EEG and EOG acquisition performed by a Brain Products amplifier (ie, LiveAmp). It is designed to provide event markers (start and end of each test) with a time precision of approximately 0.1 seconds, without requiring any programming skills. Additionally, it allows for the collection and timestamped recordings of responses to self-assessment questionnaires (KSS and CEC) and any comments the experimenter may make during the experiment.

After a preliminary configuration phase, during which the timeline for tests and questionnaires is set once and for the 4-hour block, the software has to be launched for each acquisition. It opens a graphical interface with windows on 2 separate screens (one for the experimenter and one for the participant), enabling the protocol to be run and the test and questionnaire instructions to be presented to the participant, in written and audio form, while managing the acquisition of EEG signals realized by the EEG amplifier.

The software automatically adds time markers on the fly to the protocol steps and enables the experimenter to follow the progress of the protocol via a graphical timeline and via the list of steps, to visually monitor the tracings in real time, insert customizable predefined event markers, and interrupt or resume the progress of the protocol.

### Outcome Measures

#### Primary Outcome Measure

The primary outcome measure corresponds to the OSS score, which relies on the analysis of electrophysiological variables to assess the instantaneous drowsiness state, corresponding to the participant’s level of sleepiness. The OSS score, ranging from 0 (full alertness) to 4 (very drowsy), is assigned every 20 seconds, based on specific EEG activity (amount of beta, alpha, and theta waves) observed simultaneously in 2 regions of the brain during each epoch, and accompanied by distinct eye blinks and movements (normal or slow). An OSS score of 0 corresponds to continuous beta activity, with no alpha or theta activity and no slow eye movements. In contrast, an OSS score of 4 is characterized by the presence of alpha and/or theta rhythms for more than 10 cumulative seconds, associated with slow eye movements.

The OSS was scored both manually, by a trained experimenter—based on EEG (Fz, Cz, Pz, C4, O2, C3, O1, and P3) and EOG (vertical and horizontal) recordings—and automatically, by the MEEGAWAKE algorithm—based on EEG (Cz and Pz)— every 20 seconds throughout all behavioral tests, to reflect the spontaneous drowsiness states.

The MEEGAWAKE algorithm uses data-driven criteria to cope with interindividual variability. After an initial artifact rejection step relying on detecting both temporal and frequential abrupt variations or nonphysiological behavior, the analysis step aims to determine recording-specific thresholds based on EEG power ratios in the usual frequency bands. In addition to this frequency analysis, a temporal localization of EEG events is performed to analyze burst activity. Based on the results of this analysis, the final step is to classify the 20-second epochs into OSS drowsiness states.

During MWT, sleep onset is defined as the start of the first epoch scored 3 or 4. OSS sleep latency (OSS latency) is defined as the time from lights out until the start of the first epoch scored as 3 or 4 (expressed in minutes).

#### Secondary Outcome Measures

The secondary outcome measures are the following: resting state EEG, longer resting state EEG, objective sleep quantity and quality, objective measurement of manifest sleepiness, driving performance, cognitive performance, and subjective sleepiness and mind wandering.

Resting state EEG, recorded through bio-calibrations, is a simple procedure in which participants are placed in a quiet room and asked to relax while looking at a picture on the wall, to keep their eyes open for 1 minute, refraining from blinking as much as possible, and then close their eyes for 1 minute, without moving (BC2). EEG and EOG channels are recorded continuously during bio-calibrations. In the preprocessing step of the signal analysis, standard signal processing techniques will be used, including notch filtering to remove power-line interference and bandpass filtering to isolate specific frequency subbands. The EEG data will be segmented into 4-second epochs to minimize the effects of nonstationarity in longer signal segments. Spectral power across different EEG frequency subbands and the frequency centroid will be computed for each channel under both eyes-open and eyes-closed conditions. To mitigate the influence of outliers, the median spectral power across epochs within each session will be used. EEG signal processing will be performed using the Python (Python Software Foundation) MNE toolbox (magnetoencephalography and electroencephalography in Python). Following preprocessing, feature vectors representing each session will be constructed, comprising multiple EEG-derived features. These feature vectors will then serve as inputs for a machine learning model to identify the most informative features for distinguishing participants’ levels of drowsiness, cognitive, and driving performances as quantified by the different tests conducted after the bio-calibrations.

Longer resting state EEG, recorded through a longer bio-calibration, is a procedure that remains the same as described for BC2, but here the participants are asked to keep their eyes open for 5 minutes (BC5). During the 5 minutes with eyes open, the Fz signal is analyzed in 2-second epochs, each characterized by features including the signal magnitude and spectral power (Fast Fourier Transform, Hanning window) in the frequency bands delta (0‐4 Hz), theta (4‐8 Hz), alpha (8‐12 Hz), sigma (12‐16 Hz), and beta (16‐50 Hz). After automatic artifact and blink removal using both temporal and frequential criteria, the spectral power of the artifact-free Fz signal will then be computed in the 6‐9 Hz band [57] for each 2-second artifact-free epoch. The resulting distributions of spectral power values will then be characterized by their mean, SD, and median. The mean spectral power will be the result of BC5. To compare values between individuals while controlling for interindividual variability, a participant’s test values are normalized by their median.

Objective sleep quantity and quality, measured through polysomnography recordings and analyzed by ASEEGA software (Physip), are based on an automated analysis of a single EEG channel (Cz-Pz) using a combination of multiple signal processing and classification techniques [58-60]. The preprocessing phase notably aims to adapt the analysis criteria to cope with EEG interindividual variability. The analysis step uses both frequency and temporal techniques to retrieve numerous 30-second-long and 1-second-long features as well as performing the detection of several sleep microstructural events. The classification phase then reduces and summarizes this information by scoring sleep EEG into conventional sleep stages using artificial intelligence techniques, such as pattern recognition and fuzzy logic. This method was validated in healthy individuals and demonstrated high agreement with visual scoring realized by an experienced scorer, between 82.9% and 96%, depending on the number of vigilance stages considered, allowing its application in several research studies [59,60]. Classical sleep parameters like the total sleep time (TST: the sum of all sleep stages, expressed in minutes), sleep onset latency (SOL: the time, in minutes, from lights out to the first recorded epoch of any sleep stage, N1, N2, N3, or Rapid Eye Movement), sleep efficiency (calculated as the ratio of TST to total time in bed, multiplied by 100), wake after sleep onset (calculated as the total time in bed minus the TST), and time spent in stages N1, N2, N3, and Rapid Eye Movement (expressed as a percentage of TST and in minutes) will be computed. Objective sleep quantity and quality will also be measured through actigraphy recordings and analyzed by ActiLife software (ActiGraph) using the Cole-Kripke algorithm. The sleep outcomes calculated were TST (minutes), SOL (minutes), and sleep efficiency (%).

Objective measurement of manifest sleepiness will be assessed during the MWT and analyzed visually by an experienced technician according to the AASM criteria [61]. Objective sleepiness is quantified by the SOL (minutes), defined as the time from lights out to the start of the first epoch of any sleep stage (N1, N2, N3, or rapid eye movement). If the patient succeeds in staying awake during the entire duration of the test, the SOL is set to 40 minutes.

Driving performance, assessed using a driving simulator, is quantified by the variability of the car’s lateral position (standard deviation of lateral position [SDLP; cm]), indicating weaving of the car, and the number of inappropriate crossings of lateral highway lane markers (Inappropriate Line Crossing).

Cognitive performance is assessed using the PVT, the CPT, and the TAP. For the PVT, outcome measures include mean reaction time (RT; milliseconds), the fastest and slowest 10% of RTs, and the number of lapses (RT>500 ms). For the CPT, outcomes consist of the mean RT (measuring the response execution process), the number of omissions (failure to respond to target stimuli), and commission errors (responses given to nontarget stimuli). For the TAP, outcomes include mean RT and the number of omissions (missed targets).

Subjective sleepiness and mind wandering are assessed using the KSS and the CEC, respectively. The outcome measure for both is the score on the corresponding scale.

### Data Analysis

#### Statistical Analysis

The analyses will be performed using R version 4.4.2 (R Foundation for Statistical Computing) and IBM SPSS Statistics version 27 (IBM Corp) software for Windows. The normality of the data will be assessed using the Shapiro-Wilk normality test, the equality of variance will be evaluated with the Levene test, and the sphericity of the data will be checked using the Mauchly test. Continuous variables will be expressed as means and categorical variables as proportions (%). *P* values less than .05 will be considered statistically significant.

#### OSS Criteria Accuracy Evaluation

Considering that the collected data consist of either continuous linear data, counts, or proportions, it is very likely that some of the data will not follow a normal distribution. Therefore, the use of parametric tests may be compromised. Moreover, our outcomes are measured repeatedly over time and may be highly dependent on interindividual variability. Thus, we will use a generalized linear mixed model to explore our first main objective, with relevant interindividual data incorporated into the model if needed (including patient ID, age, gender, daydreaming frequency, chronotype, and sleep history).

Using such models, OSS and MWT sleep onset latencies will be compared over time in order to determine whether OSS criteria can accurately detect drowsiness compared to the MWT.

As a second step, behavioral performance based on the OSS score will be assessed to determine whether instantaneous drowsiness states, identified by OSS, can detect momentary functional impairments. The evaluation of OSS temporal accuracy will be conducted by selecting OSS scores greater than 0 at a time T (20-second epoch), surrounded by lower score values (at T-20 seconds and T+20 seconds) [26]. Behavioral outcomes will then be compared using a generalized linear mixed model between T-20 seconds, T, and T+20 seconds of selected OSS scores.

#### Predictive Models Using Resting State EEG

To predict drowsiness-mediated behavioral performance, the proposed model leverages resting-state EEG recordings obtained prior to task execution. The primary behavioral end point is SDLP, which is dichotomized into 2 classes: “Good Performance” and “Poor Performance” based on the 33rd and 66th percentiles of the SDLP distribution. This percentile-based approach ensures approximately balanced class sizes, and the resulting thresholds and participant categorizations were validated by domain experts.

Considering the nonstationary nature of EEG signals and their susceptibility to interindividual variability, machine learning methods capable of handling such variability will be prioritized. Feature extraction and selection will be guided by SHAP (Shapley Additive Explanations) values computed on the training set. Channels and features with high importance will be selected and then evaluated on a separate validation subset within each fold. Performance will be reported on a completely unseen test set to prevent leakage.

A leave-one-subject-out cross-validation strategy will be applied to provide a robust evaluation under intersubject variability. Within each fold, to optimize hyperparameters and feature selection, a nested validation set will be defined by randomly selecting a subset of participants from the training pool (ensuring no overlap with the test participant). All preprocessing, feature selection, and hyperparameter tuning will be performed exclusively on the training and validation subsets, with the test participant held out to avoid information leakage.

Classification will be performed using CatBoost, an ensemble-based gradient boosting algorithm suitable for tabular and heterogeneous data. To enhance generalizability and benchmark the findings across distinct algorithmic frameworks, additional classifiers, including radial basis function support vector machine, decision trees, logistic regression, and extreme gradient boosting, will also be applied. Demographic and behavioral covariates (eg, age, gender, chronotype, daydreaming frequency, and sleep history) will be optionally included to account for residual interindividual variability.

Model performance will be evaluated using accuracy, precision, recall, and *F*_1_-score, reporting both macroaveraged and weighted-averaged metrics to account for potential class imbalance. Comparing these metrics allows assessment of classifier sensitivity to imbalanced classes. Bootstrapping and permutation testing will be applied to evaluate the statistical significance and robustness of the results, ensuring that predictive performance is not due to chance.

### Ethical Considerations

This study was approved by the French National Ethics Committee (Consultative Committee for the Protection of Persons participating in biomedical research, CPP Sud Est V, on April 14, 2022, under the number N° SI RIPH 2G: 22.00521.000045) and was registered in ClinicalTrials.gov (NCT05453643). The National Agency for the Safety of Medicines and Health Products was notified about this study. All staff members involved in this study ensure that the full research was conducted in accordance with ethical guidelines and regulations on research involving human participants (as stipulated in the Good Clinical Practices [International Council for Harmonisation of Technical Requirements for Pharmaceuticals for Human Use], law no. 2022‐323 of March 4, 2022, on research involving human participants, and the Declaration of Helsinki).

All participants gave their written informed consent prior to inclusion in the study. This study complies with the General Data Protection Regulation (GDPR) and the MR-001 National Commission on Informatics and Liberty reference methodology, which require data to be deidentified after collection (for privacy protection). Personal information, such as participants’ names, addresses, and medical status, will thus be exclusively managed at the examination center and not provided to third parties. All data collected were deidentified, participants being assigned a unique study code, and any information pertaining to personal details is kept in locked filing cabinets in the SANPSY laboratory, only accessible to authorized research staff directly running the study. Relevant data are entered into an electronic database using only the study codes assigned to each participant and are securely stored on an encrypted and secure server made accessible to research staff directly running the study through password-protected computers.

The risk of adverse events is considered low, and the study procedure can be canceled at any time. During the study, participants were monitored by psychologically trained staff, which ensured fast communication of complaints and an immediate response. Volunteers received financial compensation of €900 (US $1062.02) for their participation in the study. The final results of this study will be disseminated through peer-reviewed publications and conferences.

## Results

This study was funded by Physip and ANR (Agence Nationale de la Recherche, National Research Agency) in 2019, with ethical committee (Comité de Protection des Personnes, Committee for the Protection of Persons) authorization in May 2022. Candidate recruitment began in March 2023 and was completed in May 2025 (N=40), with a database lock in June 2025. Data analysis started in June 2025 and is still ongoing.

## Discussion

### Principal Findings

The objective of this study is to validate a new, easily implementable spontaneous drowsiness measurement, derived from OSS criteria and based on the automatic analysis of a limited number of EEG channels, able to reflect manifest drowsiness, particularly the ability to stay awake and the associated cognitive and behavioral consequences, regardless of the time of day. In addition, the study aims to evaluate whether iterative resting-state EEG can reliably predict drowsiness and its functional outcomes, independently of the prior duration of wakefulness. These measurements will enable the early assessment of drowsiness, allowing for the prediction of the ability to maintain wakefulness and/or of both simple and/or complex cognitive impairments associated with drowsiness.

### Effortless Installation

The key advantage of our EEG-based drowsiness detection system lies in its design for real-world applications by prioritizing a minimal number of EEG channels (ie, 2) and using short, iterative measurements. By reducing hardware complexity and optimizing data collection, our approach ensures practicality under operational conditions, making it suitable for monitoring in real-time scenarios. This approach not only enhances user comfort and system efficiency but also facilitates seamless integration into sophisticated driver and pilot monitoring systems, contributing to improved safety and performance.

### Benefits of 24-Hour EEG

As part of our study protocol, we perform continuous EEG-based monitoring of drowsiness during a 24-hour sleep deprivation period and throughout a subsequent night following daytime recovery sleep. This presents a compelling opportunity to deepen our understanding of the temporal dynamics of sleep pressure and vigilance regulation. Such long-term recordings can capture natural fluctuations in alertness due to circadian and homeostatic processes, as well as individual susceptibility to sleep loss. This comprehensive dataset could inform the development of more robust and generalizable algorithms capable of detecting drowsiness across a wide range of real-world scenarios and time frames.

Moreover, a 24-hour EEG assessment allows the identification of individual variability in drowsiness expression and recovery, potentially enabling personalized predictive models. Such models could dynamically adapt to a user’s physiological state, thereby enhancing the precision and timeliness of drowsiness detection. This approach aligns with the growing emphasis on personalized medicine and human-centered design in neurotechnology.

Developing algorithms trained on 24-hour EEG data may also support the integration of drowsiness monitoring into wearable or minimally invasive systems. By learning to identify EEG markers that remain consistent across different levels of activity, posture, and time of day, these algorithms could eventually be embedded in real-time applications, providing continuous monitoring and early warnings to mitigate fatigue-related risks.

### Resting-State EEG Predictive Advantage

Unlike conventional automatic drowsiness detection systems that identify spontaneous drowsiness or related performance declines in real-time —often too late to prevent impairments or accidents— our approach might predict drowsiness-induced performance deterioration in advance, enabling timely intervention with effective countermeasures to prevent functional decline. The aim is to assess the cognitive readiness and forecast the likelihood of either good or impaired performance before the task.

Like certain algorithms, we will leverage machine learning analyses while integrating interindividual characteristics, such as age, sex, chronotype, and accumulated sleep deprivation. This personalized approach aims to enhance predictive accuracy.

By leveraging automatic sleep pressure quantification through resting-state EEG data—adjusted for interindividual characteristics— our study will provide valuable insights into individuals’ vulnerability to drowsiness before critical lapses in attention or vigilance occur. This predictive capability enables proactive interventions, such as fatigue management strategies, to prevent performance deterioration and reduce the risk of accidents.

### Implications for the World of Work and Transport

The ability to forecast drowsiness-related impairments has significant applications in occupational and transport settings [5,62-64]. Workers in industries requiring night shifts, such as in health care, emergency response, and manufacturing, frequently experience sleep deprivation, which can lead to decreased cognitive performance and increased accident risks at work [5,65,66]. By integrating predictive drowsiness assessment tools, employers can enhance safety measures through “fatigue” monitoring and targeted interventions to prevent fatigue-related errors and accidents.

The transport sector, particularly aviation, rail, and road transport, is highly vulnerable to drowsiness-related accidents [67]. Microsleeps and lapses in vigilance can have catastrophic consequences, making early prediction of drowsiness essential for safety. Unlike real-time systems that detect spontaneous drowsiness only at the moment it occurs, our approach will offer a preemptive strategy by identifying individuals at risk of performance decline related to drowsiness beforehand. In other words, our approach systematically evaluates an operator’s cognitive readiness and forecasts the probability of optimal or impaired performance due to sleepiness prior to the initiation of a driving task, flight, or other transportation activity. Our EEG-based drowsiness detection systems can facilitate the implementation of drowsiness-monitoring technologies in pilots, train operators, and long-haul drivers, significantly reducing the likelihood of sleep-related incidents occurring. Additionally, our findings could inform regulatory policies mandating proactive drowsiness risk management in safety-sensitive professions.

Continuous real-time drowsiness assessment is essential, especially for validating Driver Drowsiness Warning systems in advanced driver-assistance systems. To detect spontaneous drowsiness during driving or work scenarios, we will develop an automated algorithm that analyzes 2 EEG channels in accordance with OSS scoring.

### Clinical Health Applications

Identifying or predicting drowsiness during behavioral or cognitive tasks will primarily be used to assess the effects of medication or other interventions in conditions related to sleepiness or in patients experiencing excessive daytime sleepiness.

Beyond occupational and transport safety, the study’s findings have important implications for clinical health. Sleep disorders, such as insomnia, sleep apnea, and narcolepsy, compromise an individual’s ability to maintain wakefulness and cognitive function. By leveraging EEG-based biomarkers to measure manifest sleepiness or predict drowsiness susceptibility, clinicians could improve diagnostic accuracy and develop more personalized treatment plans. In fact, the MWT is the reference test for measuring manifest sleepiness (ability to maintain wakefulness under monotonous conditions) in sleepy patients and to determine the efficacy of treatment for sleepiness. In some countries, the MWT has a medico-legal value and is used to determine whether an individual with sleep-related driving risk is fit or unfit to drive after the implementation of therapeutic measures. In this case, the MWT must be repeated annually for heavy vehicle licenses and every 3 years for light vehicle licenses. The MWT is conducted in specialized sleep centers and requires appropriate equipment and trained personnel for accurate execution and interpretation. However, access to these centers may be limited due to a shortage of specialists and restricted availability of necessary equipment. Automatic analysis of sleep latency using OSS criteria during the MWT can facilitate the clinical management of sleepy patients and could add, as microsleep episode, potential additional value in the context of sleepiness assessment [68]. In this sense, the freely available VIGALL (Vigilance Assessment through Graphical Analysis of Linear and Localized) signals algorithm has already demonstrated its ability to provide comparable information on wakefulness regulation as the more resource-intensive and time-consuming Multiple Sleep Latency Test [60]. However, the VIGALL requires 25 EEG derivations, which may still limit its practicality in routine clinical use compared to the MEEGAWAKE single-EEG algorithm. Moreover, automatic analysis of resting-state EEG is simpler, more cost-effective, and easier to administer, making it a potentially valuable alternative to the MWT for the initial assessment of drowsiness in clinical settings. Automatic analysis of resting-state EEG would be a simpler, more cost-effective, and easier to administer assessment, making it a potentially valuable alternative to the MWT for the initial assessment of drowsiness in clinical settings.

Additionally, older individuals, patients recovering from neurological conditions, such as traumatic brain injuries [69] or neurodegenerative diseases [70,71], often experience disrupted sleep-wake cycles and daytime sleepiness. An easy-to-administer and precise assessment of their ability to stay awake and maintain attention could enhance rehabilitation strategies, thereby improving cognitive recovery and overall well-being.

### Strengths and Weaknesses

One of the strengths of this study is its rigorous methodology, aimed at validating a drowsiness detection system through a strictly controlled sleep deprivation protocol, simulating realistic conditions of repeated night work or on-call shifts. The assessment of drowsiness throughout the sleep deprivation periods, both during the day and at night, provides a detailed understanding of its fluctuations according to circadian rhythms and the accumulation of sleep pressure. The analysis of behavioral and cognitive consequences is particularly thorough, combining subjective and objective measures through simulated driving tests, simple and complex cognitive tasks, and a scientifically recognized test for evaluating the ability to stay awake. Additionally, the sample is balanced in terms of gender and covers a wide age range (20‐60 years), enhancing the generalizability of the results to a healthy adult population.

However, this study also has some limitations. Although the laboratory setting minimizes environmental biases and ensures strict standardization of experimental conditions, it does not fully replicate the constraints and dynamics of real-world settings, particularly in professional environments. Furthermore, the relatively small sample size (N=40) limits the statistical power of the conclusions. Another notable limitation is the exclusion of patients diagnosed with sleep disorders or excessive drowsiness, restricting the applicability of the findings to healthy individuals only. As a result, it is not possible to confirm that the tested system would be equally effective in a clinical population. These limitations should be considered before broader implementation of this technology.

### Conclusion

This study is expected to contribute to a deeper understanding of the determinants of drowsiness and advance the development of proactive strategies for its monitoring and management across occupational, transport, and clinical contexts. Rather than relying solely on real-time drowsiness detection systems, this work examines how OSS-based criteria may capture spontaneous drowsiness and associated behavioral changes, and how resting-state EEG parameters may predict cognitive readiness and mid-term vulnerability to performance decline. The project aims to identify objective neuromarkers that could support earlier and more effective detection of drowsiness and sleep-related risks. Future work will involve validating these potential neuromarkers across diverse healthy and patient populations and evaluating how they may be integrated into fatigue-risk management tools and clinical assessment frameworks. Together, these efforts aim to support the broader implementation of predictive drowsiness-assessment methods for improving safety, optimizing clinical care, and reducing sleep-related accident risk.

## Supplementary material

10.2196/83969 Checklist 1 SPIRIT checklist.

## Abbreviations

AASM: American Academy of Sleep Medicine
ANR: Agence Nationale de la Recherche
CEC: Conscient Experience Characterization
CONSORT: Consolidated Standards of Reporting Trials
CPT: continuous performance test
DDFS: Daydreaming Frequency Scale
ECG: electrocardiogram
EEG: electroencephalogram
EMG: electromyogram
EOG: electrooculogram
ESS: Epworth Sleepiness Scale
GDPR: General Data Protection Regulation
INRETS-MSIS SIM2: Institut National de Recherche sur les Transports et leur Sécurité — Modélisations, Simulations et Systèmes d’Information pour la Sécurité — Simulateur version 2
KSS: Karolinska Sleepiness Scale
MSFsc: midsleep on work-free days
MSWsc: midsleep on working days
MWT: maintenance of wakefulness test
OSAS: obstructive sleep apnea syndrome
OSS: Objective Sleepiness Scale
PHQ-4: Patient Health Questionnaire-4
PVT: Psychomotor Vigilance Task
RLS: restless legs syndrome
RT: reaction time
SDLP: standard deviation of lateral position
SHAP: Shapley Additive Explanations
SOL: sleep onset latency
STOP-BANG: snoring, tiredness, observed apnea, blood pressure, BMI, age, neck circumference, and gender
TAP: Test of Attentional Performance
TST: total sleep time
VIGALL: Vigilance Assessment through Graphical Analysis of Linear and Localized
