# Supplementary material for: Evaluating Electroencephalogram-Based Predictive Model for Drowsiness Measurement to Reduce Accident Risk in Active Individuals: Protocol for a Preliminary Monocentric Study
Source: JMIR Res Protoc. 2026 Feb 17;15:e83969. doi: 10.2196/83969 (PMC12912656; doi:10.2196/83969)
Supplement: Checklist 1 [file resprot-v15-e83969-s001.pdf]

SPIRIT 2025 checklist of items to address in a randomized trial protocol*

Section / Topic	No	SPIRIT 2025 checklist item description	Reported on page no.
**Administrative information**			
Title and structured summary	1a	Title stating the trial design, population, and interventions, with identification as a protocol	4
	1b	Structured summary of trial design and methods, including items from the World Health Organization Trial Registration Data Set	4
Protocol version	2	Version date and identifier	4
Roles and responsibilities	3a	Names, affiliations, and roles of protocol contributors	4-5
	3b	Name and contact information for the trial sponsor	5
	3c	Role of trial sponsor and funders in design, conduct, analysis, and reporting of trial; including any authority over these activities	5
	3d	Composition, roles, and responsibilities of the coordinating site, steering committee, endpoint adjudication committee, data management team, and other individuals or groups overseeing the trial, if applicable	5
**Open science**			
Trial registration	4	Name of trial registry, identifying number (with URL), and date of registration. If not yet registered, name of intended registry	5
Protocol and statistical analysis plan	5	Where the trial protocol and statistical analysis plan can be accessed	5
Data sharing	6	Where and how the individual de-identified participant data (including data dictionary), statistical code, and any other materials will be accessible	5
Funding and conflicts of interest	7a	Sources of funding and other support (e.g., supply of drugs)	5
	7b	Financial and other conflicts of interest for principal investigators and steering committee members	5
Dissemination policy	8	Plans to communicate trial results to participants, healthcare professionals, the public, and other relevant groups (e.g., reporting in trial registry, plain language summary, publication)	5
**Introduction**			
Background and rationale	9a	Scientific background and rationale, including summary of relevant studies (published and unpublished) examining benefits and harms for each intervention	5-6
	9b	Explanation for choice of comparator	6
Objectives	10	Specific objectives related to benefits and harms	6
**Methods: Patient and public involvement, trial design**			
Patient and public involvement	11	Details of, or plans for, patient or public involvement in the design, conduct, and reporting of the trial	6
Trial design	12	Description of trial design including type of trial (e.g., parallel group, crossover), allocation ratio, and framework (e.g., superiority, equivalence, non-inferiority, exploratory)	6

Methods: Participants, interventions, and outcomes			
Trial setting	13	Settings (e.g., community, hospital) and locations (e.g., countries, sites) where the trial will be conducted	6
Eligibility criteria	14a	Eligibility criteria for participants	6-7
	14b	If applicable, eligibility criteria for sites and for individuals who will deliver the interventions (e.g., surgeons, physiotherapists)	7
Intervention and comparator	15a	Intervention and comparator with sufficient details to allow replication including how, when, and by whom they will be administered. If relevant, where additional materials describing the intervention and comparator (e.g., intervention manual) can be accessed	7
	15b	Criteria for discontinuing or modifying allocated intervention/comparator for a trial participant (e.g., drug dose change in response to harms, participant request, or improving/worsening disease)	7
	15c	Strategies to improve adherence to intervention/comparator protocols, if applicable, and any procedures for monitoring adherence (e.g., drug tablet return, sessions attended)	7
	15d	Concomitant care that is permitted or prohibited during the trial	7
Outcomes	16	Primary and secondary outcomes, including the specific measurement variable (e.g., systolic blood pressure), analysis metric (e.g., change from baseline, final value, time to event), method of aggregation (e.g., median, proportion), and time point for each outcome	7-8
Harms	17	How harms are defined and will be assessed (e.g., systematically, non-systematically)	8
Participant timeline	18	Time schedule of enrollment, interventions (including any run-ins and washouts), assessments, and visits for participants. A schematic diagram is highly recommended (see Figure)	8-9
Sample size	19	How sample size was determined, including all assumptions supporting the sample size calculation	9
Recruitment	20	Strategies for achieving adequate participant enrollment to reach target sample size	9
**Methods: Assignment of interventions**			
Randomization:			10
Sequence generation	21a	Who will generate the random allocation sequence and the method used	10
	21b	Type of randomization (simple or restricted) and details of any factors for stratification. To reduce predictability of a random sequence, other details of any planned restriction (e.g., blocking) should be provided in a separate document that is unavailable to those who enroll participants or assign interventions	10
Allocation concealment mechanism	22	Mechanism used to implement the random allocation sequence (e.g., central computer/telephone; sequentially numbered, opaque, sealed containers), describing any steps to conceal the sequence until interventions are assigned	10
Implementation	23	Whether the personnel who will enroll and those who will assign participants to the interventions will have access to the random allocation sequence	10
Blinding	24a	Who will be blinded after assignment to interventions (e.g., participants, care providers, outcome assessors, data analysts)	10

	24b	If blinded, how blinding will be achieved and description of the similarity of interventions	10
	24c	If blinded, circumstances under which unblinding is permissible, and procedure for revealing a participant's allocated intervention during the trial	10
**Methods: Data collection, management, and analysis**			
Data collection methods	25a	Plans for assessment and collection of trial data, including any related processes to promote data quality (e.g., duplicate measurements, training of assessors) and a description of trial instruments (e.g., questionnaires, laboratory tests) along with their reliability and validity, if known. Reference to where data collection forms can be accessed, if not in the protocol	10
	25b	Plans to promote participant retention and complete follow-up, including list of any outcome data to be collected for participants who discontinue or deviate from intervention protocols	10
Data management	26	Plans for data entry, coding, security, and storage, including any related processes to promote data quality (e.g., double data entry; range checks for data values). Reference to where details of data management procedures can be accessed, if not in the protocol	10
Statistical methods	27a	Statistical methods used to compare groups for primary and secondary outcomes, including harms	11
	27b	Definition of who will be included in each analysis (e.g., all randomized participants), and in which group	11
	27c	How missing data will be handled in the analysis	11
	27d	Methods for any additional analyses (e.g., subgroup and sensitivity analyses)	11
**Methods: Monitoring**			
Data monitoring committee	28a	Composition of data monitoring committee (DMC); summary of its role and reporting structure; statement of whether it is independent from the sponsor and funder; conflicts of interest and reference to where further details about its charter can be found, if not in the protocol. Alternatively, an explanation of why a DMC is not needed	12
	28b	Explanation of any interim analyses and stopping guidelines, including who will have access to these interim results and make the final decision to terminate the trial	12
Trial monitoring	29	Frequency and procedures for monitoring trial conduct. If there is no monitoring, give explanation	12
**Ethics**			
Research ethics approval	30	Plans for seeking research ethics committee/institutional review board approval	12
Protocol amendments	31	Plans for communicating important protocol modifications to relevant parties	12
Consent or assent	32a	Who will obtain informed consent or assent from potential trial participants or authorized proxies, and how	12
	32b	Additional consent provisions for collection and use of participant data and biological specimens in ancillary studies, if applicable	12
Confidentiality	33	How personal information about potential and enrolled participants will be collected, shared, and maintained in order to protect confidentiality before, during, and after the trial	12
Ancillary and post-trial care	34	Provisions, if any, for ancillary and post-trial care, and for compensation to those who suffer harm from trial participation	12

Administrative information		
Title and structured summary	1a	Study protocol for evaluating EEG-based predictive model for drowsiness measurement to reduce accident risk in active individuals Trial Acronym: MEEGASAFE
	1b	Trial design: preliminary monocentric study that modify the level of drowsiness by varying the prior sleep duration and the timing of sleep within 24 hours.<br>Methods: The duration and quality of sleep (EEG, EOG, EMG) will be recorded either in the laboratory or ambulatory, and analyzed manually according to AASM criteria, as well as automatically using the ASEEGA algorithm. Drowsiness will be objectified through EEG recording and manually analyzed according to the OSS criteria, and also automatically analyzed using the MEEGAWAKE algorithm. The ability to stay awake will be measured by the maintenance of wakefulness test (MWT). Subjective sleepiness will be measured before and after all measurements. Several cognitive tasks will be performed every 4 hours to measure sustained attention, vigilance, and selective attention. Driving performance will be analyzed every 4 hours in the context of a monotonous highway driving scenario conducted on a driving simulator.<br>Primary registry and trial identifying number: CPP Sud Est V N° ID-RCB: 2021-A03234-37<br>Date of registration in primary registry: 01/02/2022<br>Source(s) of monetary or material support: ANR<br>Public title: EEG analysis for objective assessment of drowsiness to limit accidental risk<br>Countries of recruitment: France<br>Health condition(s) / problem(s) studied: Healthy subjects / accidental risk linked to drowsiness<br>Intervention(s): Sleep quantity modifications<br>Key inclusion and exclusion criteria: see item 14a<br>Date of first enrolment: 08/03/2023<br>Target sample size: 40<br>Recruitment status: completed<br>Outcome(s): see item 16
Protocol version	2	14/04/2022 Version: 1.2
Roles and responsibilities	3a	Chloé Boitard[1], Zoé Mazurie[1], Khadijeh Sadatnejad[1], Julien Coelho[1], Patricia Sagaspe[2], Julie Lenoir[1], Julien Mattei[3], Pierre Berthomier[3], Marie Brandwinder[4], Pierre Philip[2], Jean-Arthur Micoulaud Franchi[2], Christian Berthomier[3], Jacques Taillard[1,5]*<br><br>[1] Univ. Bordeaux, SANPSY, UMR 6033, Bordeaux, France<br>[2] CHU de Bordeaux, Service Universitaire de Médecine du Sommeil, Bordeaux, France,<br>[3] Physip, Paris, France<br>[4] ENSAM, Paris, France<br>[5] CNRS, SANPSY, UMR 6033, Bordeaux, France<br>* Corresponding author (ORCID 0000-0001-9067-8189)

		- Chloé Boitard (PhD): data curation, formal analysis, visualization, writing – original draft preparation, writing – review & editing.<br>- Khadijeh Sadatnejad (PhD): formal analysis, methodology, software, writing – original draft preparation, writing – review & editing.<br>- Zoé Mazurie (PhD): formal analysis, writing – review & editing.<br>- Julien Coelho (PhD, MD): formal analysis, writing – review & editing.<br>- Patricia Sagaspe (PhD): methodology, writing – review & editing.<br>- Julie lenoir (PhD): writing – review & editing.<br>- Julien Mattei (Engineer): data curation, software.<br>- Pierre Berthomier (MSc): data curation, software.<br>- Marie Brandewinder (PhD): Funding acquisition.<br>- Pierre Philip (PhD, MD): project administration, writing – review & editing.<br>- Jean-Arthur Micoulaud Franchi (PhD, MD): Principal Investigator, writing – review & editing.<br>- Christian Berthomier (PhD): conceptualization, formal analysis, funding acquisition, methodology, software, supervision, writing – original draft preparation, writing – review & editing.<br>- Jacques Taillard (PhD): conceptualization, funding acquisition, methodology, supervision, visualization, writing – original draft preparation, writing – review & editing.
	3b	Agence Nationale de la Recherche (ANR) 86 rue Regnault 75013 PARIS.
	3c	This funding source had no role in the design of this study and will not have any role during its execution, analyses, interpretation of the data, or decision to submit results.
	3d	Not applicable.
**Open science**		
Trial registration	4	French national ethical committee (Comité de Protection des Personnes Sud Est V) N° ID-RCB: 2021-A03234-37.
Protocol and statistical analysis plan	5	https://clinicaltrials.gov/study/NCT05453643
Data sharing	6	All relevant deidentified data will be made available upon study completion and results publication.
Funding and conflicts of interest	7a	This work was supported by the National Research Agency (ANR) under Grant (ANR-19-LCV1-0004 MEEGASAFE), received by J.Taillard and C. Berthomier.
	7b	There are no financial and no competing interests for principal investigators for the study.
Dissemination policy	8	The final results will be disseminated through peer-reviewed publications and conferences.
**Introduction**		
Background and rationale	9a	Voluntary behaviors and socio-economic factors, such as social jetlag and shift work, can lead to insufficient or disrupted sleep, resulting in drowsiness in active individuals. In occupational and driving contexts, drowsiness poses a serious safety risk by impairing alertness, slowing reaction times, and increasing the likelihood of accidents. Developing automatic and easy to implement

		tools for drowsiness detection or prediction is essential in sleepy patient management or in high-risk environments where sustained vigilance is critical. This study aims to validate a continuous or predictive methods for assessing drowsiness using automated analysis of a limited number of electroencephalogram (EEG) channels. Designed as single-center, non-randomized, single-group, this study will evaluate drowsiness and cognitive performance in forty healthy volunteers exposed to two sleep deprivation conditions simulating real-world occupational scenarios. The primary outcome will be the Objective Sleepiness Scale (OSS) and its automated analysis, with a focus on its ability to measure objective wakefulness as assessed by the Maintenance of Wakefulness Test (MWT). Secondary outcomes will include multimodal resting-state EEG markers, subjective and objective sleepiness measures, performance on a simulated driving task, attention, executive function and vigilance assessments, as well as sleep quality, sleep quantity, and mind-wandering. The influence of sociodemographic and clinical variables on drowsiness measurement and prediction will also be systematically examined. By validating these novel EEG-based measures, this study aims to lay the groundwork for proactive drowsiness management strategies in occupational, transportation and clinical settings.
	9b	Not applicable.
Objectives	10	This study has two main objectives. First, the aim is to determine whether Objective Sleepiness Scale (OSS) criteria can accurately detect manifest sleepiness, as compared to the Maintenance of Wakefulness Test (MWT), and to evaluate its temporal accuracy in detecting drowsiness-induced momentary behavioral outcomes, including driving performance, vigilance, and sustained and selective attention.<br>Second, this study aims to validate robust models based on a multimodal EEG index, derived from resting-state activity during bio-calibration, for predicting functional impairments linked to drowsiness, particularly in driving performance, vigilance, sustained and selective attention.
**Methods: Patient and public involvement, trial design**		
Patient and public involvement	11	Healthy subjects, patients and the public were not involved in the design, conduct, reporting, or dissemination plans of this trial.
Trial design	12	Preliminary single-group, single-center research. Modification of the drowsiness level by varying the duration of prior sleep within 24 hours.
**Methods: Participants, interventions, and outcomes**		
Trial setting	13	Bordeaux University hospital (monocentric); data will only be collected in this institution (France).
Eligibility criteria	14a	The eligibility criteria include the following inclusion criteria: subjects aged between 20 and 60, with a body-mass index between 18 and 27, having good French skills and ability to understand the study, and being non-professional drivers with valid driver license (obtained at least one year ago). Subjects suffering from severe psychiatric, neurological or medical pathology, or under psychotropic or cardiotropic drug treatments, subjects suffering from chronic insomnia disorder, severe diurnal somnolence or sleeping pathologies that can induce excessive daytime

		somnolence, subjects declaring substance dependency, alcohol abuse (> 2 glasses/day) and/or excessive consumption of coffee, tea or caffeine-based drinks (such as coke, > 5 cups/day), and subjects that perform night or shift work being on care or on-call duty during the last 72 hours before the experimental sessions, are excluded from the study.
	14b	Not applicable.
Intervention and comparator	15a	All subjects will be placed in sleep deprivation patterns corresponding to sleep deprivation generated by work schedules usually observed (night work or night on-call duty) to modify the state of secondary hypersomnolence. On-call work will be simulated by two half-hour induced awakenings during the night, and hypersomnolence will be measured in the 12 hours of wakefulness following this night of sleep fragmentation. Night work will be simulated by a full sleep deprivation for 24 hours, followed by a 4h morning sleep, and a prolonged wakefulness for 19 hours. Sleep history (quantity and quality of sleep) will be assessed by actimetry + ambulatory polysomnography in the night prior to experimentation. Experimentation will begin by a night in the laboratory with a polysomnography. Ambulatory recordings will be analysed automatically by the ASEEGA algorithm (developed by Physip). Polysomnography recordings will be analysed visually and automatically by ASEEGA. Throughout the experiment, electrophysiological variables (EEG, EOG and EMG) will be continuously recorded to identify secondary hypersomnolence states, determined visually using OSS or automatically by the MEEGAWAKE algorithm (developed by Physip). Maintenance of wakefulness tests (MWT), driving simulation and cognitive tests will be repeated to objectivize abilities to stay awake, driving performance, sustained and selective attention and vigilance throughout the sleep deprivation patterns.
	15b	Not applicable.
	15c	Not applicable.
	15d	Not applicable.
Outcomes	16	<u>Primary outcome</u>:<br>- Sleepiness states classified according to the OSS score, based on analysis of electrophysiological variables (EEG, EOG and EMG).<br><u>Secondary outcomes</u>:<br>- Resting state EEG, registered through bio-calibrations (EEG, EOG).<br>- Sleep quantity and quality. Classical sleep parameters (total sleep time (min), sleep onset latency (min), sleep efficiency (%), quantity of N1, N2, N3 and R stages (min and %), wakefulness time after sleep onset (min), latency to persistent sleep (min) will be calculated using actimetry, polysomnographic recording (minimum 2 EEGs, maximum 6 EEGs, 1 EMG and 2 EOGs) performed in ambulatory and in the laboratory.<br>- Measurement of manifest sleepiness, assessed during the Maintenance of Wakefulness Test (MWT) and quantified by the sleep onset latency. This test lasts a maximum of 40 minutes.

		- Driving performance, measured on the INRETS-MSIS SIM2 driving simulator. Subjects will drive for 20 minutes on a freeway. The number of inappropriate line crossings and the variability of the vehicle's lateral position in the lane will be calculated.<br>- Sustained attention, assessed by the Simple Reaction Time Test (PVT). Outcome measures include mean reaction time (RT, in milliseconds), the fastest and slowest 10% of RTs (in milliseconds, and the number of lapses (RT > 500 ms). This test lasts 10 minutes.<br>- Vigilance, assessed by the “vigilance” TAP. Outcomes include mean RT and the number of omissions (missed target). This test lasts 30 minutes.<br>- Sustained and selective attention assessed by CPT II (Continuous Performance Test, CPT). Outcomes consist of the mean RT, the number of omission (failure to respond to target stimuli) and commission errors (responses given to nontargets stimuli). The test lasts 15 minutes.<br>- Subjective sleepiness and mind wandering, assessed by the Karolinska Sleepiness Scale (KSS) and the Conscient Experience Characterization (CEC), respectively.
Harms	17	At all times there will be contact to psychologically trained staff, which ensures fast communication of complaints and immediate response. Furthermore, the study procedure can be cancelled at any time. The occurrence probability of further risks is estimated low.
Participant timeline	18	Time schedule of enrolment, assessments, and visits for participants. This study does not include any interventions.

	Study period				
	Interview	Enrolment	Experimental schedule		Close-out
**Timepoint**	$T_{-1} = T_0 - 1$ to 15 days	T0	Session 1 $T_1 = T_0 + 1$ to 14 days	Session 2 $T_2 = T_1 + 7$ to 30 days	$T_3 = T_2 + 4$ days
**Recruitment**					
Information about the study	X				
Eligibility screen	X	X			
Informed consent		X			
Clinical examination		X			
Inclusion self-questionnaires (PHQ-4, ESS, STOP-BANG, RLS)		X			
Individual characteristics (chronotype, daydreaming)		X			
**Assessments**					
Actimetry			X	X	
Polysomnography			X	X	
Continuous wake EEG recordings (OSS, resting state EEG)			X	X	
Karolinska Sleepiness Scale			X	X	
Driving simulation			X	X	
Maintenance of Wakefulness Test			X	X	
Cognitive tests			X	X	
Conscient Experience Characterization			X	X	

Sample size	19	This study is exploratory, since to our knowledge, no other study has used OSS to predict manifest sleepiness. Thus, because no data could allow us to statistically determine the number of subjects required, this number has arbitrarily been set to 40 healthy volunteers.
Recruitment	20	Recruitment will take place by means of the Bordeaux University Hospital's healthy volunteer database, and by advertisement via information-flyers and internet recruitment.
**Methods: Assignment of interventions**		

Randomization:		
Sequence generation	21a	Not applicable.
	21b	Not applicable.
Allocation concealment mechanism	22	Not applicable.
Implementation	23	Not applicable.
Blinding	24a	Not applicable.
	24b	Not applicable.
	24c	Not applicable.
**Methods: Data collection, management, and analysis**		
Data collection methods	25a	Procedures in this study are standardized test procedures and questionnaires (see 12 for more details). Driving performance and cognitive tests outcomes are directly generated by the software. Scores on self-evaluation questionnaires are provided by the subject. Data are reported by a trained assessor. Ambulatory recordings will be analysed automatically by the ASEEGA algorithm (developed by Physip). Polysomnography recordings will be analysed manually by a trained assessor and automatically by ASEEGA. Electrophysiological variables (EEG, EOG and EMG) recorded throughout the experiment to allow identification of sleep phases and drowsiness states will be scored visually by a trained assessor (using OSS) and automatically by the MEEGAWAKE algorithm (developed by Physip).
	25b	Subjects will receive a financial compensation (900€) for participating to the whole study. Subjects abandoning the study before the end of will not be considered, and will be replaced, until obtention of 40 healthy volunteers that fulfilled the whole protocol.
Data management	26	All data gathered in the context of the study exclusively serve research purposes, are kept in confidence and deitentified. Participant data are generated directly by the software (cognitive and driving test performance, EEG recordings) or by the subject (scores on self-evaluation questionnaires) and are entered by the clinical research associate in the subject's pseudo-anonymized observation notebook (identified only by the subject's code, consisting of an identification number and initials), stored securely in a locker on the SANPSY research platform. Data is transferred to the computer database, stored on the Bordeaux University Hospital's secure, encrypted server. Only authorized personnel conducting the research may consult these files. The concordance between the information provided by the software, recorded in the observation book and copied into the Meegasafe computer database is verified by double reading. More details of data management procedures can be found in the DMP Opidor (Projcet "Analyse de l’EEG pour l’évaluation de la somnolence afin d’améliorer la sécurité" https://dmp.opidor.fr/plans/29938/export.pdf?export%5Bquestion_headings%5D=true)

Statistical methods	27a	The analyses will be performed using R 4.4.2 and IBM SPSS Statistics v. 27 (IBM Corp., Armonk, N.Y., USA) software for Windows. The normality of the data will be assessed using the Shapiro-Wilk normality test, the equality of variance will be evaluated with Levene's test, and the sphericity of the data will be checked using Mauchly's test. Continuous variables will be expressed as means and categorical variables as proportions (%). P-values less than 0.05 will be considered statistically significant.<br><br><u>OSS criteria accuracy evaluation</u>:<br>The outcomes are measured repeatedly over time and are potentially highly dependent on inter-individual variability. Thus, we will use a Generalized Linear Mixed Model (GLMM) to explore our first main objective, with relevant inter-individual data incorporated into the model if necessary (including patient ID, age, gender, daydreaming frequency, chronotype, and sleep history). OSS and MWT sleep onset latencies will be compared over time, in order to determine whether OSS criteria can accurately detect drowsiness, compared to the MWT.<br>As a second step, behavioral performance based on the OSS score will be assessed, to establish whether instantaneous drowsiness states, identified by OSS, can detect momentary functional impairments. The evaluation of OSS temporal accuracy will be done by selecting OSS scores greater than 0 at a time T (20-second epoch), surrounded by lower score values (at T-20 s and T+20 s) [62]. Behavioral outcomes will then be compared using GLMM between T-20 s, T and T+20 s of selected OSS scores.<br><br><u>Predictive models using resting state EEG</u>:<br>To predict drowsiness-mediated behavioral performance, the proposed model will leverage resting-state EEG recordings obtained prior to task execution. Behavioral outcomes will be categorized into two classes, "Good Performance" and "Poor Performance", based on the 33rd and 66th percentiles of the behavioral performance distribution. Machine learning methods capable of handling EEG signals variability are prioritized. Following the identification of EEG-based predictive neuromarkers and their relative contributions using explainable artificial intelligence (XAI) techniques, classification will be performed using the Categorical Boosting (CatBoost) algorithm. A leave-one-subject-out (LOSO) cross-validation strategy will be applied to ensure robust evaluation under inter-subject variability conditions. Support Vector Machine with a Radial Basis Function kernel (RBF-SVM), will be employed as a complementary model to the ensemble-based CatBoost approach. If required, demographic and behavioral covariates (i.e. age, gender, chronotype, daydreaming frequency, sleep history) will be incorporated into the models.
	27b	All participants will be included in each analysis.
	27c	Not applicable.
	27d	Not applicable.

Methods: Monitoring		
Data monitoring committee	28a	DMC is not needed because of known minimal risks.
	28b	Not applicable.
Trial monitoring	29	No data monitoring committee was established for this study. As this is a non-interventional study classified as RIPH 2 (research involving minimal risks and constraints under French ethical regulations), the level of risk for participants was considered minimal, and therefore no formal data monitoring was deemed necessary. Study conduct was overseen by the coordinating investigator and the sponsor through regular supervision of study progress, adherence to the protocol, and verification of data collection procedures.
**Ethics**		
Research ethics approval	30	Ethics approval was obtained from the French Ethics Committee (consultative Committee for the Protection of Persons participating in biomedical research, CPP sud est V, under the number N° SI RIPH 2G: 22.00521.000045). Moreover, the study is registered ClinicalTrials.gov (number NCT05453643) and the National Agency for the Safety of Medicines and Health Products was notified about this study.
Protocol amendments	31	Not applicable.
Consent or assent	32a	The principal investigator will obtain informed consent in written form from all study participants.
	32b	Not applicable.
Confidentiality	33	For privacy protection, participants will be identified using an identification code. Information such as participants' names and addresses will be managed exclusively at the examination center and will not be provided to third parties. Experimental data necessary to provide to a joint research institution will be carefully protected using only the participant's identification codes.
Ancillary and post-trial care	34	Not applicable.

*We strongly recommend reading this checklist in conjunction with the SPIRIT 2025 Explanation and Elaboration and the SPIRIT 2025 Expanded Checklist for important clarifications on all the items. We also recommend reading relevant SPIRIT extensions. See www.consort-spirit.org

Citation: Chan A-W, Boutron I, Hopewell S, Moher D, Schulz KF, et al. SPIRIT 2025 statement: updated guideline for protocols of randomised trials. BMJ 2025;389:e081477. https://dx.doi.org/10.1136/bmj-2024-081477

© 2025 Chan A-W et al. This is an Open Access article distributed under the terms of the Creative Commons Attribution License (https://creativecommons.org/licenses/by/4.0/), which permits unrestricted use, distribution, and reproduction in any medium, provided the original work is properly cited.
